# Linoleate-pazopanib conjugation as active pharmacological ingredient to abolish hepatocellular carcinoma growth

**DOI:** 10.3389/fphar.2023.1281067

**Published:** 2024-01-16

**Authors:** Ke Wang, Pei-Yin Liao, Wei-Chun Chang, Cian-Ru Yang, Yu-Ting Su, Ping-Ching Wu, Yang-Chang Wu, Yao-Ching Hung, Najim Akhtar, Hsueh-Chou Lai, Wen-Lung Ma

**Affiliations:** ^1^ Graduate Institute of Biomedical Sciences, and Ph.D. Program for Health Science and Industry, School of Medicine, China Medical University, Taichung, Taiwan; ^2^ Department of Medical Research, Chinese Medicine Research and Development Center, and Department of Obstetrics and Gynecology, China Medical University Hospital, Taichung, Taiwan; ^3^ Department of Biomedical Engineering, National Cheng Kung University, Tainan, Taiwan; ^4^ Institute of Oral Medicine and Department of Stomatology, National Cheng Kung University Hospital, College of Medicine, National Cheng Kung University, Tainan, Taiwan; ^5^ Center of Applied Nanomedicine, National Cheng Kung University, Tainan, Taiwan; ^6^ Medical Device Innovation Center, Taiwan Innovation Center of Medical Devices and Technology, National Cheng Kung University Hospital, National Cheng Kung University, Tainan, Taiwan; ^7^ Graduate Institute of Integrated Medicine, College of Chinese Medicine, China Medical University, Taichung, Taiwan; ^8^ Department of Obstetrics and Gynecology, Asia University Hospital, Taichung, Taiwan; ^9^ Center for Digestive Medicine, Department of Internal Medicine, China Medical University Hospital, Taichung, Taiwan

**Keywords:** hepatocellular carcinoma, LDC, linoleate, pazopanib, LAPC

## Abstract

Small molecule compounds targeting multiple kinases involved in neoangiogenesis have shown survival benefits in patients with unresectable hepatocellular carcinoma (HCC). Nonetheless, despite the beneficial effects of multikinase inhibitors (MKIs), a lack of boosting adjuvant limits their objective response rate. Lipid conjugates have been used to improve delivery efficacy or pharmaceutical benefits for decades. However, the feasibility of utilizing lipid-drug conjugates (LDCs) in HCC regimens remains untested. In this study, oral feeding of linoleate-fluorescein isothiocyanate conjugates showed that the compound was well distributed in a spontaneous HCC mouse model. Therefore, a rationale design was developed for chemically synthesizing a linoleate-pazopanib conjugate (LAPC). The LAPC showed a significantly improved cytotoxicity compared to the parental drug pazopanib. Pazopanib’s angiogenic suppressing signals were not observed in LAPC-treated HCC cells, potentially suggesting an altered mechanism of action (MOA). In an efficacy trial comparing placebo, oral pazopanib, and LAPC treatments in the hepatitis B virus transgene-related spontaneous HCC mouse model (HBVtg-HCC), the LAPC treatment demonstrated superior tumor ablating capacity in comparison to both placebo and pazopanib treatments, without any discernible systemic toxicity. The LAPC exposure is associated with an apoptosis marker (Terminal deoxynucleotidyl transferase dUTP nick end labeling [TUNEL]) and an enhanced ferroptosis (glutathione peroxidase 4 [GPX4]) potential in HBVtg-HCC tumors. Therefore, the LAPC showed excellent HCC ablative efficacy with altered MOA. The molecular mechanisms of the LAPC and LDCs for HCC therapeutics are of great academic interest. Further comprehensive preclinical trials (e.g., chemical-manufacture-control, toxicity, distribution, and pharmacokinetics/pharmacodynamics) are expected.

## 1 Introduction

Liver cancer is the fifth most common malignancy in men and the ninth in women worldwide. It is also the second most common cause of cancer mortality (Chidambaranathan-Reghupaty et al., 2021). Hepatocellular carcinoma (HCC) is the most common liver cancer type (Xie, 2017). It is a highly aggressive cancer, which usually develops from long-term chronic liver inflammation and injury. Its major risk factors are chronic infections with hepatitis B virus (HBV) or hepatitis C virus, alcohol use, carcinogens, and inherited diseases (Suresh et al., 2020; Mahmud et al., 2021). The prognosis of individuals diagnosed with HCC remains suboptimal, with a 5-year survival rate of 18% (Villanueva, 2019). Targeting cancer-associated driver mutations is an ideal strategy for controlling HCC, in addition to surgical resection, radiotherapy, and chemotherapy (Ding et al., 2017). Due to the rich neo-vasculature pattern of HCC (Anisha et al., 2021), many angiogenesis-targeting Multikinase Inhibitor (MKIs) have been developed (Rahbari et al., 2016). Clinically, only a few inhibitors with limited efficacy, such as sorafenib (Stra et al., 2021), lenvatinib (Lee et al., 2022), regorafenib (Iavarone et al., 2021) and cabozantinib (Kelley et al., 2022), were employed. However, the overall survival rate of patients with advanced HCC is low and has not improved (Toyoda et al., 2022). The first approved systemic therapy drug for advanced HCC (sorafenib) displayed a low objective response rate (ORR) of 2%–3% in patients (Llovet et al., 2008; Cheng et al., 2009). The second drug approved for the initial treatment of advanced HCC (Lenvatinib) demonstrated a significantly higher ORR (18.2% vs. 4.5%, *p* = 0.020) and disease control rate (77.3% vs. 47.7%, *p* = 0.001) compared to sorafenib (Choi et al., 2022). The second-line treatment with regorafenib improved the overall survival of patients with HCC who showed disease progression during first-line treatment with sorafenib (Bruix et al., 2017). Cabozantinib has been granted approval for patients with advanced HCC treated with sorafenib and is currently being developed in combination with immune checkpoint inhibitors in patients (Trojan 2020). Despite numerous advances, therapeutic responses to MKIs vary widely in individual patients and across patient populations (Huang et al., 2020). These results may be attributable to the potency and selectivity of MKI, the variability in drug metabolism and pharmacokinetics, tumor biology, and the tumor microenvironment (Wedam et al., 2006). Therefore, preventing, decreasing, or reversing resistance remains a major bottleneck in HCC therapy, impeding improvements in morbidity and mortality (Chaffer and Weinberg, 2011).

As stated above, the main limitation of MKIs in HCC treatment is insufficient ORR. Recent studies have attempted to improve drug delivery in order to overcome the ORR problem. Lipid-drug conjugates (LDCs) were developed to increase drug delivery (Markovic et al., 2019). As an illustration, a phospholipid-drug conjugate (CLR 131) demonstrated an ORR of 34.5% in patients with multiple myeloma and 42% in patients with non-Hodgkins lymphoma (de Lartigue, 2020). In general, the basic design concept of LDCs was to conjugate a drug with a fatty acid (FA), glyceride, or phospholipid in order to modify its lipophilicity (Bui et al., 2019; Vishwakarma et al., 2019). The lipophilic modification of drugs can significantly alter their physical and chemical properties, improve their lipophilicities and abilities to be entrapped by lipid carriers, and increase their transmembrane abilities, improving their delivery pharmacokinetics and pharmacodynamics (Du et al., 2018; Ghasemiyeh and Mohammadi-Samani, 2018). Secondly, lipophilic modification introduces lipid ligands into their structures, which is more conducive to recognizing various lipid-related receptors on cell membranes (Ma et al., 2017; Managuli et al., 2018; Signorell et al., 2018). Thirdly, lipophilic modification has the potential to enhance drug stability, circumvent the first-pass effect, and improve bioavailability (Kaithwas et al., 2017). In addition, LDCs have advantages in targeting tumor cells, enhancing efficacy, and reducing side effects and toxicity (He et al., 2017; Irby et al., 2017). LDCs have become one of the hotspots in current pharmaceutical research and have impressive application prospects (Luo et al., 2016; Andonova and Peneva, 2017).

In this study, we employ the LDC design concept to modify MKIs and synthesize a novel FA-conjugating drug to inhibit HCC in mouse models therapeutically. MKIs conjugation with FAs may change drugs’ molecular pharmacodynamics or pharmacokinetics *in vivo*, as well as their toxicity profile. Other advantages of a drug designed to fuse with FA include increased oral bioavailability, enhanced tumor targeting effectiveness, controlled drug release, and increased cellular penetration, all of which contribute to enhanced therapeutic efficacy (Fattahi et al., 2020). Linoleic acid (LA), the most common polyunsaturated FAs (PUFAs) in nature, has been reported to engage in both pro- and anti-cancer activities (Xu and Qian, 2014). In the field of antitumor drug design, LA has been frequently combined with drugs to increase their lipid solubility (Cheng et al., 2019; Uwaezuoke et al., 2022). Herein, a linoleate-pazopanib conjugate (LAPC) was synthesized and its antitumor effects were tested both *in vivo and in vitro*.

## 2 Materials and methods

### 2.1 Reagents

All chemicals in reaction were analytical grade, purchased from Sigma-Aldrich (St. Louis, MO, United States), Alfa Aesar (Ward Hill, MA, United States), and Merck (Darmstadt, Germany). The purity of compounds was determined by TLC plates coated with Merck Silica gel 60 F254 (0.2 mm). Spots were observed under UV lamp or stained by dyeing agent. Linoleic acid (LA) was purchased from Merck; Pazopanib was purchased from GSK. ^1^H-NMR and ^13^C-NMR spectra were recorded on Bruker Avance 400 MHz spectrophotometer (Billerica, MA, United States). In 1H-NMR, CDCl3 was used as d-solvent, TMS as internalstandard to mark 0 ppm. The definition of splitting term: singlet (s), doublet (d), triplet (t), qutratet (q), multiplet (m), coupling constant (*J*). In ^13^C-NMR, chloroform was used as internal standard to mark 77.0 ppm. Mass Spectroscopy (MS) and High-Resolution Mass Spectroscopy (HRMS) were recorded on JMS-700 (JEOL), (Tokyo, Japan), double focusing mass spectrometer (FAB and EI), Applied Biosystems 4,800 Proteomics Analyze (MALDI) (Foster City, CA, United States) or Waters (Milford, MA, United States) LCT Premier XE (ESI). Dulbecco’s Modified Eagle Medium (DMEM) and Penicillin-Streptomycin were purchased from Gibco (Waltham, MA, United States). Fetal Bovine Serum (FBS) was purchased from HyClone (Marlborough, MA, United States). Methythiazolyltetrazolium (MTT) was purchased from Sigma-Aldrich. Culture insert for migration assay was purchased from ibidi (Planegg, Germany). Anesthesia for animal test (Zoletil) was purchased from Virbac (Carros, France). Fluorescence interpretation and image analysis were collected using Cytation™ 5 Cell Imaging Multi-Mode Reader, BioTek (Winooski, VT, United States). Rabbit anti-VEGFR antibody (Cell signaling, #2479), rabbit anti-Phospho-VEGFR antibody (Cell signaling, #2478), rabbit anti-PI3K antibody (Cell signaling, #4249), rabbit anti-PLCg1 antibody (Cell signaling, #2822), rabbit anti-Phospho-PLCg1 antibody (Cell signaling, #2821), rabbit anti-AKT antibody (Cell signaling, #4685), rabbit anti-Phospho-AKT antibody (Cell signaling, #4060), rabbit anti-ERK antibody (Cell signaling, #4695), rabbit anti-Phospho-ERK antibody (Cell signaling, #4370), mouse anti-b-actin antibody (Santa Cruz, sc-47778), secondary antibody: Goat anti-mouse IgG-HRP antibody (Santa Cruz, sc-2005), Goat anti-rabbit IgG-HRP antibody (Santa cruz, sc-2004).

### 2.2 FITC labeling of linoleic acid

The fluorescent compound FITC has a thio-cyanate group, which contributes to its sensitizing properties and is capable of reacting with amines and thiol residues. With gentle stirring, FITC solution in DMSO (5 mg/mL) was added dropwise to LA solution (15 mg/mL) in DMSO with the molar ratio of FITC:LA varying to 1:1. Continuous slow stirring was performed for 0.5–4 h at room temperature, protecting the mixture from light. The FITC-LA conjugates were lyophilized overnight. Then the lyophilized FITC-LA powder was stored at −20°C for further use.

### 2.3 Chemistry

The synthesis of linoleoyl pazopanib (LAPC): To a solution of pazopanib hydrochloride (0.24 g, 0.50 mmol) in DMF (20.00 mL), EDC (0.31 g, 1.50 mmol), DMAP (0.37 g, 3.00 mmol) and linoleic acid (0.18 mL, 0.55 mmol) were added and the mixture was stirred at room temperature for 7 days. The residue was evaporated under reduced pressure, treated with water (20 mL) and extracted with DCM (3 × 20 mL). The organic phase was dried (Na_2_SO_4_), evaporated under reduced pressure and purified by column chromatography using DCM:MeOH = 30:1 as eluent. White solid (250.0 mg, 0.36 mmol); yield: 71.29 percent.

^1^H-NMR (500 MHz, CDCl_3_) δ: 0.87 (t, 3H), 1.20–1.33 (m, 16H), 1.97–2.04 (m, 4H), 2.33 (t, 2H), 2.60 (s, 3H), 2.64 (s, 3H), 2.74 (t, 3H), 3.62 (s, 3H), 4.12 (s, 3H), 5.29–5.35 (m, 4H), 5.83 (d, 1H), 6.82 (t, 1H), 7.16 (d, 1H), 7.46 (s, 1H), 7.47 (d, 1H), 7.62 (d, 1H), 7.79 (d, 1H), 8.77 (s, 1H), 8.89 (s, 1H).

^13^C-NMR (125 MHz, CDCl_3_) δ: 162.8, 156.4, 150.1, 147.6, 141.7, 138.2, 137.7, 132.6, 132.3, 130,6, 130.2, 130.0, 127.9, 127.8, 123.8, 121.9, 120.9, 120.2, 119.8, 114.7, 97.1, 39.3, 37.5, 36.6, 31.9, 31.6, 31.5, 31.3, 29.7, 29.5, 29.4, 29.3, 29.2, 29.1, 29.0, 28.7, 28.6, 27.4, 27.2, 25.6, 25.2, 24.9, 24.7, 24.6, 22.7, 22.5, 22.4, 19.7.

HRMS (ESI): m/z [M + H]^+^ calcd for C_39_H_54_N_7_O_3_S: 700.4009, found: 700.4012; m/z [M - H]^-^ calcd for C_39_H_52_N_7_O_3_S: 698.3852, found: 698.3857.

### 2.4 Cell lines and cell culture

HCC cell lines (HepG2, Huh7, HCC36 and Tong) were seeded in 96 well plates (1×10^4^ cells/well) and incubate at 37°C for overnight. The cells were treated with the indicated drug dose (0, 100, 200, 400 μM and 800 μM) for 48 h. After treatment, the culture medium was added with 10 μL Cell Proliferation Reagent WST-1 (1:10 final dilution) at 37°C for 1 h 1 h later, the colorimetric absorbance of cells at 490 nm were recorded.

### 2.5 Western blot

Cells were lysed by ice-cold RIPA lysis buffer including protease inhibitor cocktail (25x, Roche) for 1 h. Samples were centrifuged for 30 min at 11,000 rpm at 4°C and the supernatants were harvested. About 50 ug of each protein sample were loaded and separated on 8% and 10% SDS gel and transferring to 0.45 mm PVDF membrane. The membranes were then blocked with 2.5% BSA for 1 h, probed with primary antibodies at a dilution of 1:1,000, followed incubation by HRP-conjugated secondary antibodies at a dilution of 1: 5,000.

### 2.6 Mouse model and treatment procedures

All of the animal experiments followed the Guidance of the Care and Use of Laboratory Animals of the National Institutes and Health and approved by China Medical University (CMUIACUC-2022-002). For LA-FITC treatment, HBVtg-HCC mouse model (Wu et al., 2010) was induced by intraperitoneal injection of low dose of DEN (20 mg/kg body weight) in 10 to 14-day-old pups (Zheng et al., 2007). Oral administration of LA-FITC three times a week at 32 weeks to 35 weeks and then the liver tumor foci was evaluated at 36 weeks of sacrifice. For placebo, pazopanib, and LAPC treatment, HBVtg-HCC mouse also induced by DEN injection. The drug was dissolved in sunflower oil and PBS mixture buffer (1:1:8) and fed 10 mg/kg of drug to mice at 42 weeks to 45 weeks and the body weight, liver weight, and tumor foci were evaluated at 46 weeks of sacrifice.

### 2.7 Immunohistochemistry (IHC)

Mouse liver tissue were fixed by 10% formalin and embedded into paraffin following standard protocol. The tissue sections were stained with primary antibodies against CD34 (1:500; ab81289, abcam) and GPX4 (1:100; sc-166570, Santa Cruze) by using an ABC kit (Vector Laboratories) to enhance the staining signals. TUNEL assay: the sections were cut from paraffin-embedded mouse liver tissues to detect cell death by using *In Situ* Cell Death Detection Kit (Roche, United States) followed the manufacturer’s instructions.

### 2.8 Statistical analysis

Analyses were performed in triplicate, and the results were expressed as mean ± SD. Analysis of variance (ANOVA) was conducted, followed by Dunnett’s *post hoc* test, to determine significant (*p* < 0.05) differences. Statistical analyses were performed using GraphPad Prism v8.0 (GraphPad Software, San Diego, CA, United States).

## 3 Results

### 3.1 LA conjugation localizes a fluorescence moiety in tumor lesions of a spontaneous HCC mouse model

**FIGURE 1:**
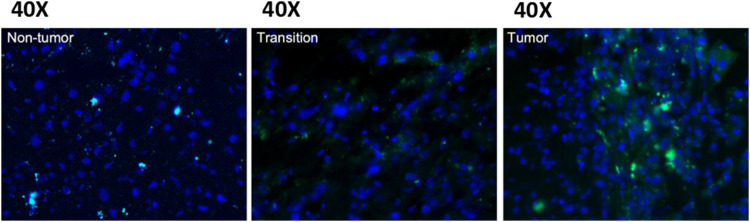
The fluorescence intensity of LA-FITC in a spontaneous HCC mouse model. FITC fluorescence is more intense in tumor tissues than non-tumor and transition tissues when viewed under a 40× microscope. This observation indicates that the LA is more likely to enter tumor tissues than other tissues.

To provide a proof-of-concept for using LDCs, we conjugated LA, a long-chain FA that cannot be synthesized by human cells, with fluorescein isothiocyanate (FITC) and fed it to a hepatitis B virus transgene-related spontaneous HCC (HBVtg-HCC) mouse model via oral gavage. We then observed the distribution of FITC in tissues (Figure 1). FITC was predominantly detected in liver tumors, with a rare incidence observed in normal adjacent or parental lesions. These data showed that FITC delivery to the tumor was effective via lipid conjugation, indicating that LA, as a type of PUFA, is more readily taken up by tumor tissues than other tissues, potentially reflecting differences in FA metabolism between cancer and normal cells. Consequently, LA could be an excellent carrying entity for HCC treatment.

### 3.2 Rationale for LA-conjugated MKIs

**FIGURE 2:**
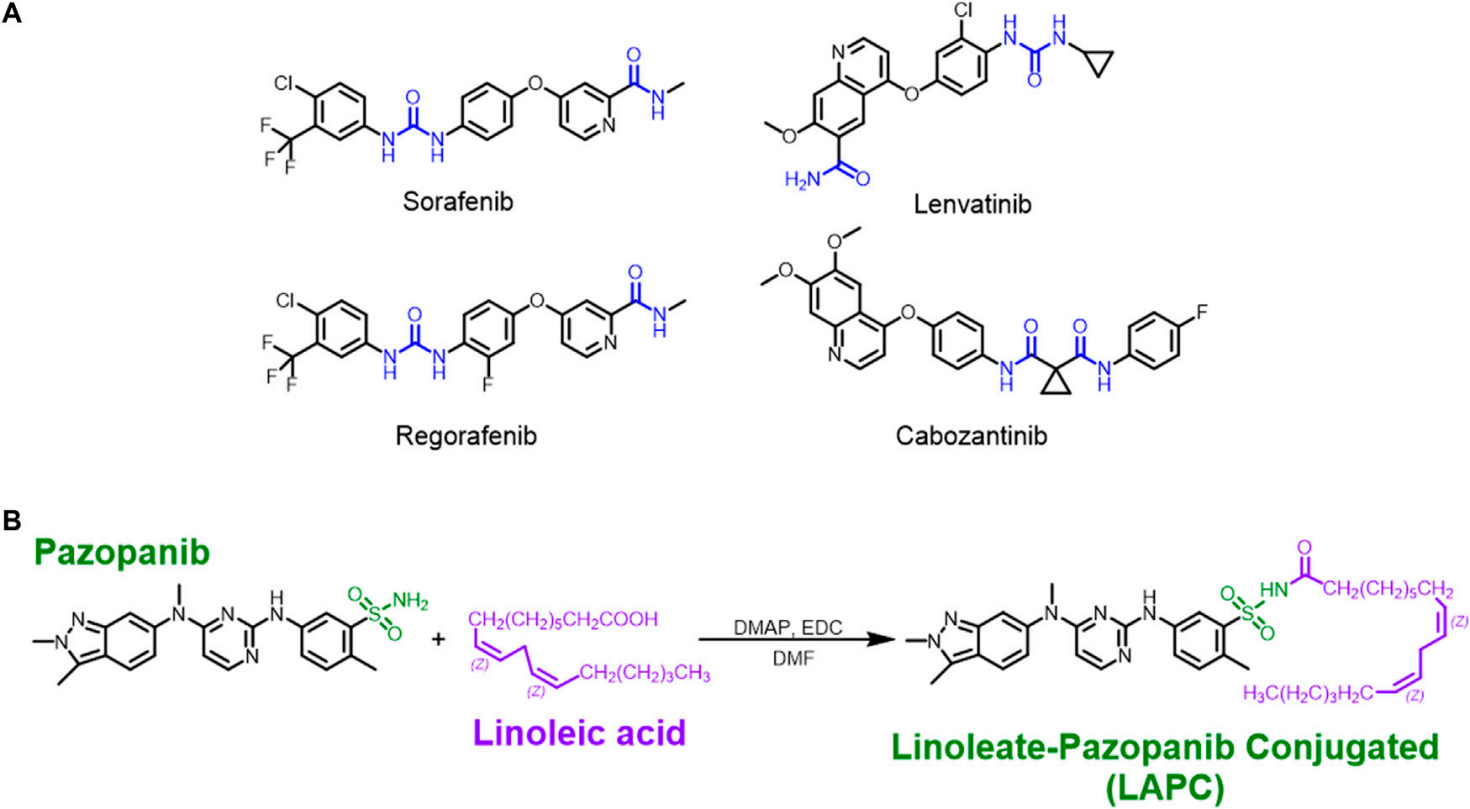
The structures of MKIs and synthesis of LA + pazopanib to produce LAPCs. **(A)** The amide group (blue part) could react with EDC under mild conditions. The structures of inhibitors were broken under the strenuous NaH condition. **(B)** The method to synthesize LAPC. The sulfonamide group (green part) provided a free amino group for the mild reaction without breaking the pazopanib structure.

Figure 2A shows several common MKIs. In our study, we first tried to add lipids to N-containing groups in the lenvatinib structure. However, the amide’s (blue part) amino group was greatly influenced by the oxygen atom, decreasing its nucleophilicity so that a mild 1-ethyl-3-(3-dimethyl-aminopropyl)-carbodiimide (EDC) reaction could not occur. Then, we used sodium hydride (NaH) to enhance the alkalinity of the reaction and make the amino group more nucleophilic. The reaction failed since a fragmented structure was observed by nuclear magnetic resonance (NMR; Supplementary Figures S1–S4). However, fragmented structures were also found in reactions of regorafenib conjugated with FA, indicating that the harsh conditions caused the reactions to fail (Supplementary Figures S5–S8). Therefore, we had to choose MKIs with a structure with a free amine group. Sorafenib and cabozantinib have similar structures to lenvatinib and regorafenib (amide group, blue part), potentially indicating difficulty in the reaction with FA. Another clinically used MKI, pazopanib (Figure 2B), was chosen because its sulfonamide group (green part) has a free amino group. This structure provided an excellent basis for chemically synthesizing pazopanib conjugated to an FA (e.g., LA).

To practice the concept of an LDC with pazopanib, we conjugated LA onto pazopanib to create a new chemical entity (LAPC) for treating HCC. The chemical synthesis procedure is shown in Figure 2B. The synthetic design concept was that nitrogen atoms are present in most MKI structures and form polar and nucleophilic functional groups. We have used PUFAs since their nucleophilic N-containing groups can couple with an aliphatic acid to install a hydrophobic residue on various anticancer drugs. In our study, pazopanib and LA were conjugated using a reaction containing EDC and 4-dimethylaminopyridine dissolved in dimethylformamide. This reaction created the LAPC product with a high yield and no regioisomer. Altogether, these results showed that our synthetic protocol was efficient for achieving high regio-selectivity, and it is unnecessary to perform multi-step chemical modifications or use an orthogonal protection strategy. The experimental procedure was simple and only required extraction and recrystallization. The new compound was confirmed by ^1^H-NMR (Supplementary Figure S9), ^13^C-NMR (Supplementary Figure S10), and mass spectrometry (Supplementary Figures S11, S12).

### 3.3 LAPC improves parental drug anti-cancer efficacy with an altered MOA

**FIGURE 3:**
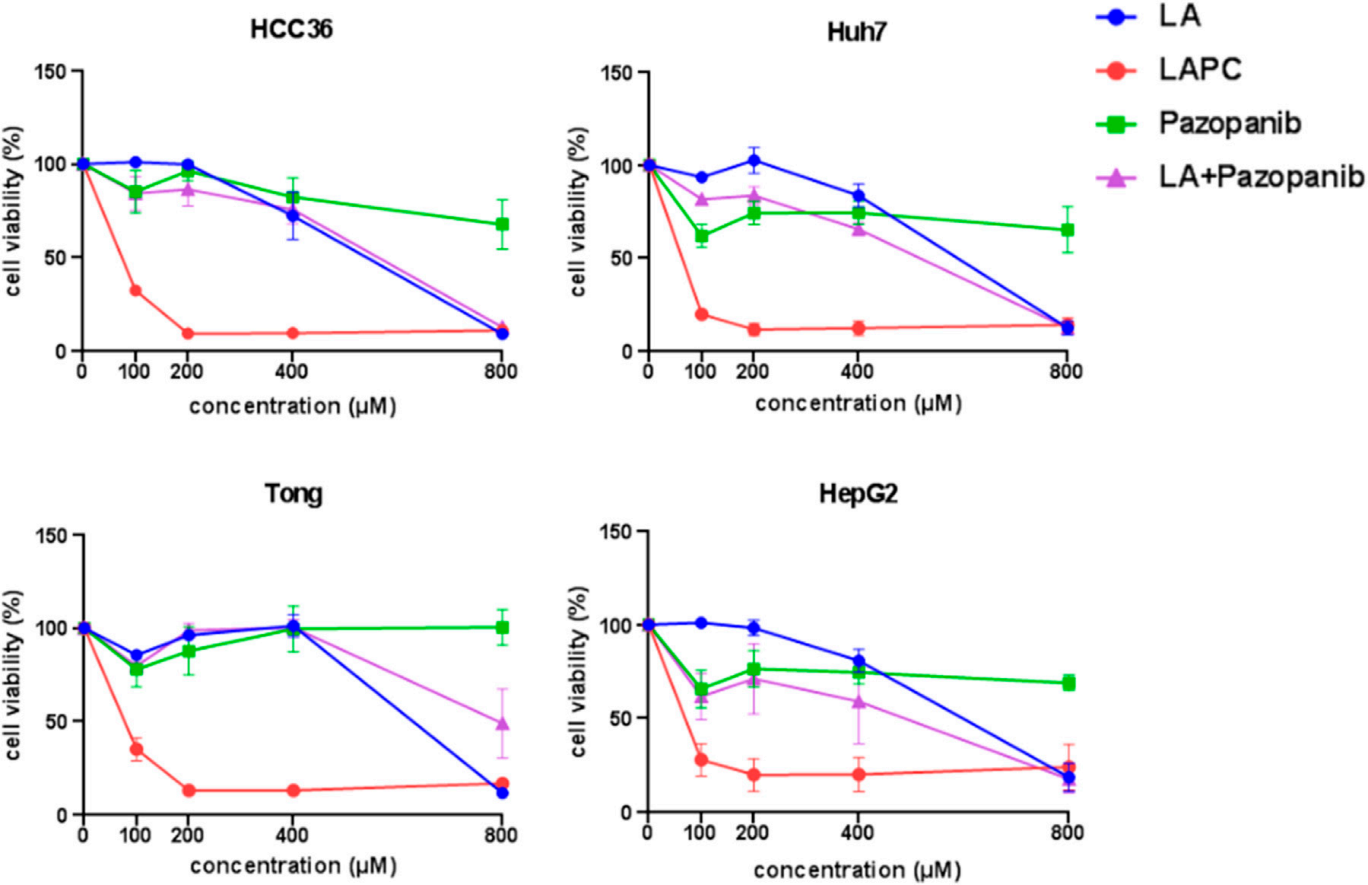
Effects of LAPCs on the viability of Huh7, Tong, HCC36, HepG2, and HepRG cells. The cells were treated with different LA, LAPC, pazopanib, and LA with pazopanib (LA + pazopanib) concentrations for 48 h. Cell viability was measured using the 3-(4,5-dimethylthazolk-2-yl)-2,5-diphenyl tetrazolium bromide (MTT) assay.

**FIGURE 4:**
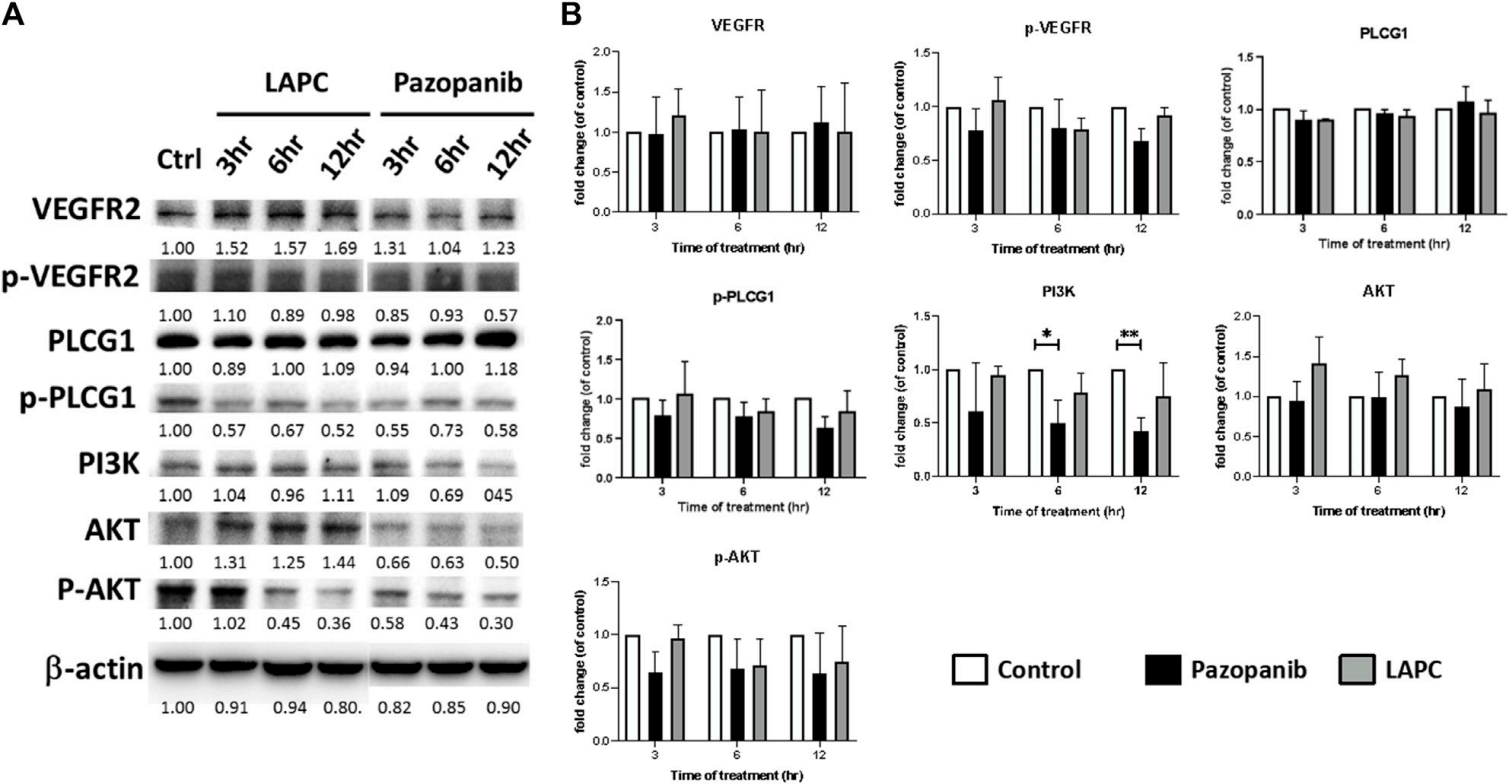
**(A)** Western blot analysis of LAPC and pazopanib effects on angiogenesis-related signaling in HCC36 cells. Equal protein loading was confirmed by reprobing the membrane with β-actin after stripping. **(B)** Quantification of angiogenesis signals. The relative levels of all proteins are reported as means ± standard deviations (SD) across triplicate samples for each treatment.

The mechanism of action (MOA) of MKIs is to suppress cellular growth signals. This inhibition prevents the activation of cellular signals related to angiogenesis (e.g., Ras/Rac, mitogen-activated protein kinase [MEK]/extracellular signal-regulated kinase [ERK], phosphoinositide 3-kinase [PI3K]/protein kinase B [AKT], and phospholipase C gamma 1 [PLCG1]) that are responsible for cancer cell growth and proliferation. Preclinical evaluations of pazopanib’s MOA showed it suppresses Ras/Rac, MEK/ERK, and PI3K/AKT activity by inhibiting VEGFR in the nanomolar range (Sonpavde and Hutson, 2007; Bukowski et al., 2010). However, its *in vitro* cytotoxic efficacy is astonishingly high in HCC cells (Zhu et al., 2011). Therefore, this study assessed whether the LAPC retained the same pharmacological characteristics when treating HCC cells by measuring its cytotoxicity (Figure 3 and Table 1) and related signaling (Figure 4).

**TABLE 1:** *In vitro* inhibitory effect of LA, LAPC, Pazopanib and LA/Pazopanib cotreatment in HCC cells.

Compound	48 h cytotoxicity (IC50 µM)^a^			
	HCC36^b^	Huh7^b^	Tong^b^	HepG2^b^
LA	>500	>500	>500	>500
LAPC	19.6	50.2	40.1	54.3
Pazopanib	>500	>1,000	>1,000	>1,000
LA/Pazopanib	454.4	>1,000	>500	337.7

^a^ Inhibition of cell growth by listed compounds was determined using MTT, assay.

^b^ HCC36, Huh, Tong and HepG2 are human HCC, cell lines.

To investigate the effects of LAPC and pazopanib on HCC cell growth, we exposed Huh7, Tong, HCC36, and HepG2 cells to five concentrations (0, 100, 200, 400, and 800 μM) of LA, LAPC, pazopanib, and LA + pazopanib for 48 h (Figure 3). The MTT assay results showed that LAPC provided better inhibition than the parental drug pazopanib, LA, and LA + pazopanib. LA had no inhibitory effect at 0–200 μM but did have an inhibitory effect at 400–800 μM. Pazopanib had little effect on HCC cellular toxicity and did not show synergy when combined with LA. The LA + pazopanib cotreatment showed cellular toxicity at high concentrations (400–800 μM). Table 1 shows the four treatments’ half-maximal inhibitor concentrations (IC50) in the various cell lines. The LAPC had an IC50 of 19.6–54.3 μM, while LA and pazopanib had IC50s of >500 μM in all cell lines. The LAPC had a greater anti-proliferative effect on the HCC36 cell line than the other treatments.

The discrepant cytotoxicity between LAPC and pazopanib motivated us to investigate the expression levels of total and phosphorylated (p) VEGFR, PLCG1, AKT, and ERK forms in HCC36 cells. As an MKI, pazopanib showed a good inhibitory effect on the levels of phosphorylated signaling proteins. However, interestingly, the LAPC had little effect on the phosphorylation of the examined signaling proteins. PLCG1 plays an important role in anti-apoptotic signaling in cells (Jang et al., 2018; Lu et al., 2020). PI3K/AKT is an intracellular signaling pathway that regulates the cell cycle and is associated with cellular quiescence, proliferation, cancer, and longevity (King et al., 2015). The pazopanib treatment resulted in a downregulation of p-PLCG1, p-AKT, and the expression of PI3K, indicating a potential inhibition of tumor cell growth and possible mechanisms via apoptosis. However, p-PLCG1, pAKT, and p-ERK levels were comparable when cells were treated with the LAPC. Altogether, these results suggest that the LAPC may have a different MOA than pazopanib.

### 3.4 The LAPC shows excellent tumor suppression efficacy without general toxicity in the HCC mouse model

**FIGURE 5:**
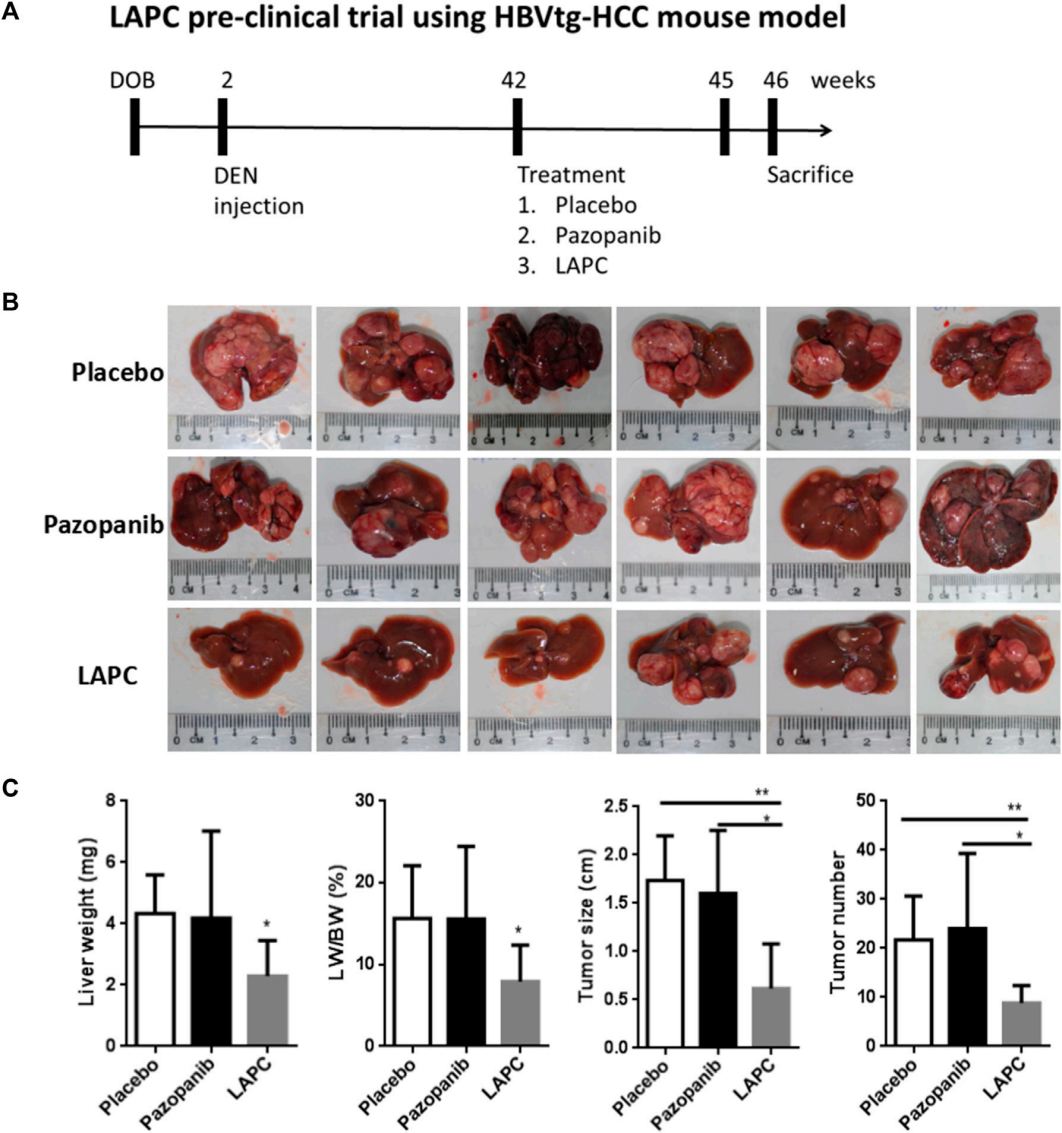
The LAPC had better therapeutic efficacy than pazopanib in the HBVtg-HCC mouse model. HBVtg-HCC mice were induced with a subminimal diethylnitrosamine injection. The drug was dissolved in a sunflower oil and phosphate-buffered saline mixture (1:1:8) and fed to 42–45-week-old mice thrice weekly at 10 mg/kg. **(A)** The LAPC preclinical trial using HBVtg-HCC mouse model. **(B)** Tumor images with a ruler as the reference. **(C)** Treatment statistics (e.g., LW, LW/BW, and tumor size and number). The data are expressed as the mean ± standard error of the mean (SEM; *n* = 6). Key: *, *p* < 0.05 compared to the LAPC group.

Since we had successfully synthesized the LAPC, we wanted to compare its therapeutic efficacy to various compounds using the spontaneous HCC mouse model. We applied them in the HBVtg-HCC mouse model (Figure 5A) and monitored various tumor parameters, including liver weight (LW) and its ratio to body weight (LW/BW), tumor size (the diameter of the largest liver tumor), and tumor number (number of tumor nodules in the liver). The tumor’s appearance is shown in Figure 5B. While measuring body weight during therapy, the LAPC was well tolerated, and no body-weight loss was observed compared to other treatments at a dosage of 20 mg/kg. The LAPC induced greater tumor inhibition than the placebo and parental pazopanib treatments (tumor growth inhibition rate = 51.7% at 20 mg/kg). The LAPC also decreased liver weight and the liver/body ratio. Moreover, tumor sizes and numbers were also lower with LAPC treatments than with the other treatments. These results suggest that LA conjugation enhanced pazopanib’s therapeutic efficacy, possibly by redistributing it to liver tumors.

### 3.5 The LAPC increased cell ferroptosis in the HBVtg-HCC mouse model

**FIGURE 6:**
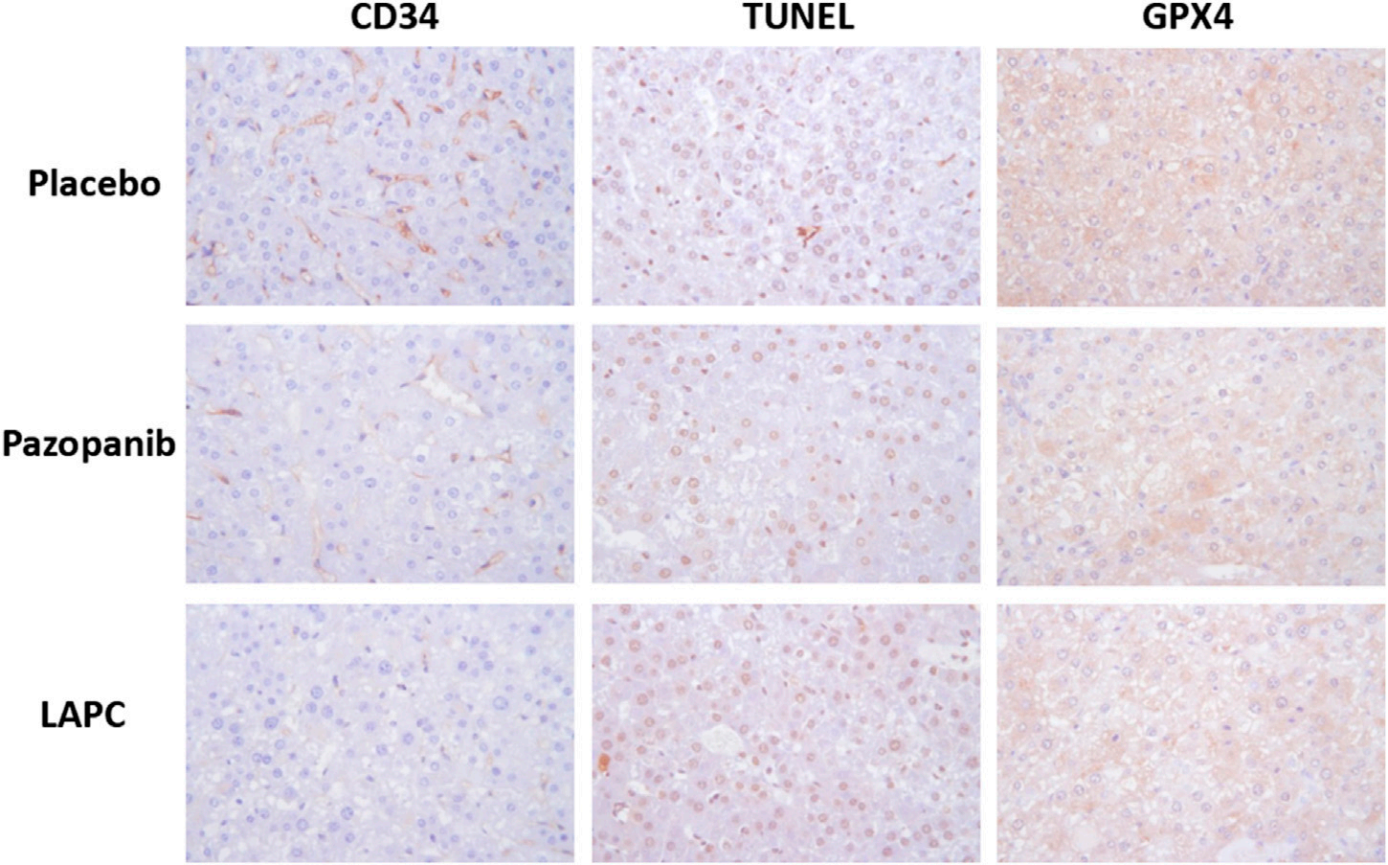
Immunohistochemical protein detection in tumor tissues. Mouse liver tissue was fixed in 10% formalin and embedded in paraffin following a standard protocol. Immunohistochemical staining was performed, with proteins appearing brown. CD34 (angiogenesis marker) and GPX4 (ferroptosis marker) inhibition were observed with pazopanib and LAPC treatments. However, TUNEL (apoptosis marker) staining did not differ significantly. Original magnification = ×100. Scale bar = 200 μm.

We hypothesized that several cellular events might underlie the MOA of LAPC-mediated HCC suppression (e.g., angiogenesis, cell apoptosis or lipid-related death, and ferroptosis). We explored these hypotheses using immunohistochemical staining for markers of the cell vascular endothelium (cluster of differentiation 34 [CD34]), apoptosis (terminal deoxynucleotidyl transferase dUTP nick-end labeling [TUNEL]), and ferroptosis (glutathione peroxidase 4 [GPX4], a phospholipid hydroperoxidase against membrane lipid peroxidation; Figure 6). CD34 staining was abundant after the placebo treatment but reduced after pazopanib and LAPC treatments. TUNEL staining was comparable after the placebo and LAPC treatments but reduced after the pazopanib treatment. GPX4 staining was lower after pazopanib treatment, particularly LAPC treatment, than the placebo treatment.

Therefore, the data in Figure 6 indicates that the LAPC might maintain some of the MKI characteristics in increasing cell apoptotic death but also enhance some rarely reported cellular death mechanisms (e.g., ferroptosis).

## 4 Discussion

In this study, the LAPC showed better therapeutic efficacy *in vitro* and *in vivo*. We discuss our findings from the following perspectives.

### 4.1 Pharmaceutical perspectives of pazopanib and LAPC dosing form

Pazopanib was designed to be administered orally, making it a convenient option for patients with difficulty undergoing intravenous chemotherapy or other invasive treatments. Pazopanib is converted to hydrochloride form to increase its stability in the digestive tract. While pazopanib had very little *in vitro* toxicity in our data, it suppressed advanced renal cell carcinoma in patients (Sternberg et al., 2013; Cella and Beaumont, 2016; Rhee et al., 2017). The MOA reported by GSK was via suppression of growth signals and anti-angiogenic activity (Kumar et al., 2007; Deng et al., 2013), not through cytotoxicity. The LAPC’s IC50 ranged from 20 to 100 μM, showing dramatic improvement compared to its parental drug. We treated the HBVtg-HCC mouse model orally with pazopanib and the LAPC (10 mg/kg/time), finding that the LAPC had a much superior therapeutic benefit. Compared to GSK’s preclinical trial, the effective dosing in mice was within 50–100 mg/kg/daily via intravenous injection (Kumar et al., 2007). It was very obvious that the LAPC showed excellent improvement in the oral dosing form. Due to LA’s esterification, pazopanib’s properties and distribution changed. LAPC could reach its target more efficiently, decreasing metabolism and bodily elimination.

In summary, LDC could improve biological utilization because esterified drugs typically have longer blood retention and lower toxicity. LDC could also alter the drug’s solubility and crystalline structure, improving its biological activity and stability. Therefore, esterifying pazopanib to LAPC is a potential drug design approach that could be used in the research and development of VEGFR inhibitors to potentially improve their biological properties, efficacy, and safety.

### 4.2 The LAPC’s possible MOA

In the MTT assay, the LAPC showed an inhibitory effect in HCC cells (Figure 3). At first, we hypothesized that conjugating pazopanib with LA may increase its lipophilicity, leading to more moderate apoptosis than pazopanib (Zhu et al., 2011). However, in the Western blot analysis (Figure 4), while pazopanib inhibited HCC cell growth through an anti-apoptotic related pathway (e.g., AKT and PLCG1), the LAPC did not. Importantly, the LAPC showed much better tumor inhibitory effects than pazopanib in the mouse model (Figure 5), indicating a different MOA *in vivo*. Immunohistochemical staining (Figure 6) showed that the LAPC decreased GPX4 expression, indicating possible ferroptosis. Altogether, this unexpected result may be due to the introduction of a PUFA into pazopanib’s structure. In addition to the possible distribution of lipid-enhanced drugs in the tumor area, the unsaturated double bonds on long-chain FAs are possibly easily oxidized in cells and undergo lipid peroxidation causing ferroptosis (Yang et al., 2016). After being inactivated by the LAPC, GPX4 cannot prevent the oxidation of a large fraction of PUFAs, leading to the accumulation of lipid peroxides and promoting the cell into ferroptosis.

### 4.3 Feasibility of chemical reaction for pharmaceutics

MKIs have been developed in the last two decades. They have significantly improved patient survival and quality of life, and shifted the treatment paradigm for various solid tumors (Huang et al., 2020). It is an insufficient ORR that limits HCC therapeutic efficacy (Sennino et al., 2012). As new MKIs are continually invented for drug development, our data provide a new strategy for their structural modification. Besides combining with traditional cytotoxic drugs, combining with other kinase inhibitors (e.g., immune checkpoint inhibitors) may be a possible approach for cancer treatment (Kato et al., 2019). Compared to single-target drugs or drug combinations, MKI drugs can overcome the limitations of single-target drugs by attacking multiple cancer hallmarks. They simultaneously achieve robust and durable therapeutic effects and avoid the risks associated with multicomponent drug cocktails, such as unpredictable pharmacokinetic profiles, drug-drug interactions, and poor patient compliance (Anighoro et al., 2014). A few examples of direct structural modifications to VEGFR inhibitors to obtain multitarget drugs exist. Zang et al. discovered a novel pazopanib-based dual inhibitor targeting cancer epigenetics and angiogenesis (Zang et al., 2018). Their study indicated that the pazopanib indazole moiety fit well into VEGFR2’s inside pocket and that the 2-aminopyrimidine moiety formed two important hydrogen bonds with Cys917 in the hinge region. Therefore, the modification of these two moieties could not be tolerated. In contrast, the benzenesulfonamide was directed toward the solvent region, where it could be modified with other chemical groups. In our study, we synthesized LA to benzenesulfonamide based on this concept, leading to a good result.

### 4.4 Potential of LDC for developing nanoparticle drugs

A liposome nanoparticle is a drug vehicle for encapsulating chemical entities or biosimilars. Due to the nature of LDC, the LAPC could be in the form of a liposome. Triglyceride and phospholipid could be chosen as the conjugated lipid, allowing the LDCs to potentially self-assemble. Self-assembling prodrugs are an emerging class of therapeutic agents that can spontaneously associate into well-defined supramolecular nanostructures in aqueous solutions (Wang et al., 2020). The key characteristic of self-assembling prodrugs is that they are amphiphilic, possessing both hydrophilic and hydrophobic domains that enable aqueous assembly (Palao-Suay et al., 2016). LDC has recently gained the attention of bionanotechnologists due to its amphiphilicity, an ideal characteristic for forming stable self-assembled nanostructures in water (Zhong et al., 2016). Diverse LDC-based nanomedicines have been developed and shown to significantly improve biological efficacy, such as an oligomer chain of ethylene glycol-camptothecin (Shen et al., 2010) and squalenoyl-doxorubicin (Maksimenko et al., 2014). LA-based drug conjugates have also been nanoformulated, with LA-conjugated paclitaxel (PTX) self-assembled into nanostructures via the precipitation method (Zhong et al., 2016). These LA-PTX nanocomposites were stable for >9 months and showed ∼2-fold higher anti-cancer ability than free LA-paclitaxel conjugates. By self-assembling into well-defined nanostructures, the resultant assemblies have a distinct, often improved pharmacokinetic profile and may have unique properties for tuning drug release rates and addressing multidrug resistance (Cheetham et al., 2017).

Besides self-assembled nanoparticles, LDCs can also be incorporated within lipid-based nanoparticles as one element of the lipid phase. A recent study used conjugated LA with cocoa butter (10:1 w/w) as the lipid phase for synthesizing a Ploxamer 407-stabilized nanostructured lipid carrier (Hashemi et al., 2020).

Furthermore, LDCs can also be used as a drug compound in nanocarriers. Since the molecular weight of FAs in the LDC is lower than polymers, the drug occupancy rate and loading efficiency into the nanocarrier are superior for LDCs (Zhong et al., 2016). One such study by Cheng (Cheng G et al., 2019) loaded SN38 conjugated LA into a polymeric nano-matrix. Additionally, LDCs improve the physicochemical properties of drug molecules for nanoformulation, such as aqueous solubility and surface functionality. In this study, LAPC showed higher hydrophobic than free pazopanib, which can be beneficial for loading into organic solvent-friendly nanocarriers. We hope that the successfully LDC synthesis described in this article will improve the feasibility of drug development for the treatment of liver cancer.

## 5 Conclusion

The LDC approach demonstrated in this article has four main advantages: (1) Its chemical synthesis of LAPC is easy for pharmaceutical development without regioisomers; (2) Its robust yield via simple extraction and recrystallization at room temperature keeps production cost down; (3) It significantly improved therapeutic efficacy compared to the parental drug, first demonstrated in HCC; (4) Its concept of conjugating therapeutic lipids with small molecule drugs is feasible, allowing greater possibilities for pharmaceutical development. Overall, this study might pave the way for novel cancer therapies. Further detailed pharmacological studies are needed.

## Data Availability

The original contributions presented in the study are included in the article/Supplementary Material, further inquiries can be directed to the corresponding authors.
