# Supplementary material for: Linoleate-pazopanib conjugation as active pharmacological ingredient to abolish hepatocellular carcinoma growth
Source: Front Pharmacol. 2024 Jan 16;14:1281067. doi: 10.3389/fphar.2023.1281067 (PMC10824963; doi:10.3389/fphar.2023.1281067)
Supplement: Supplementary file 1 [file DataSheet1.docx]

Supplementary Material


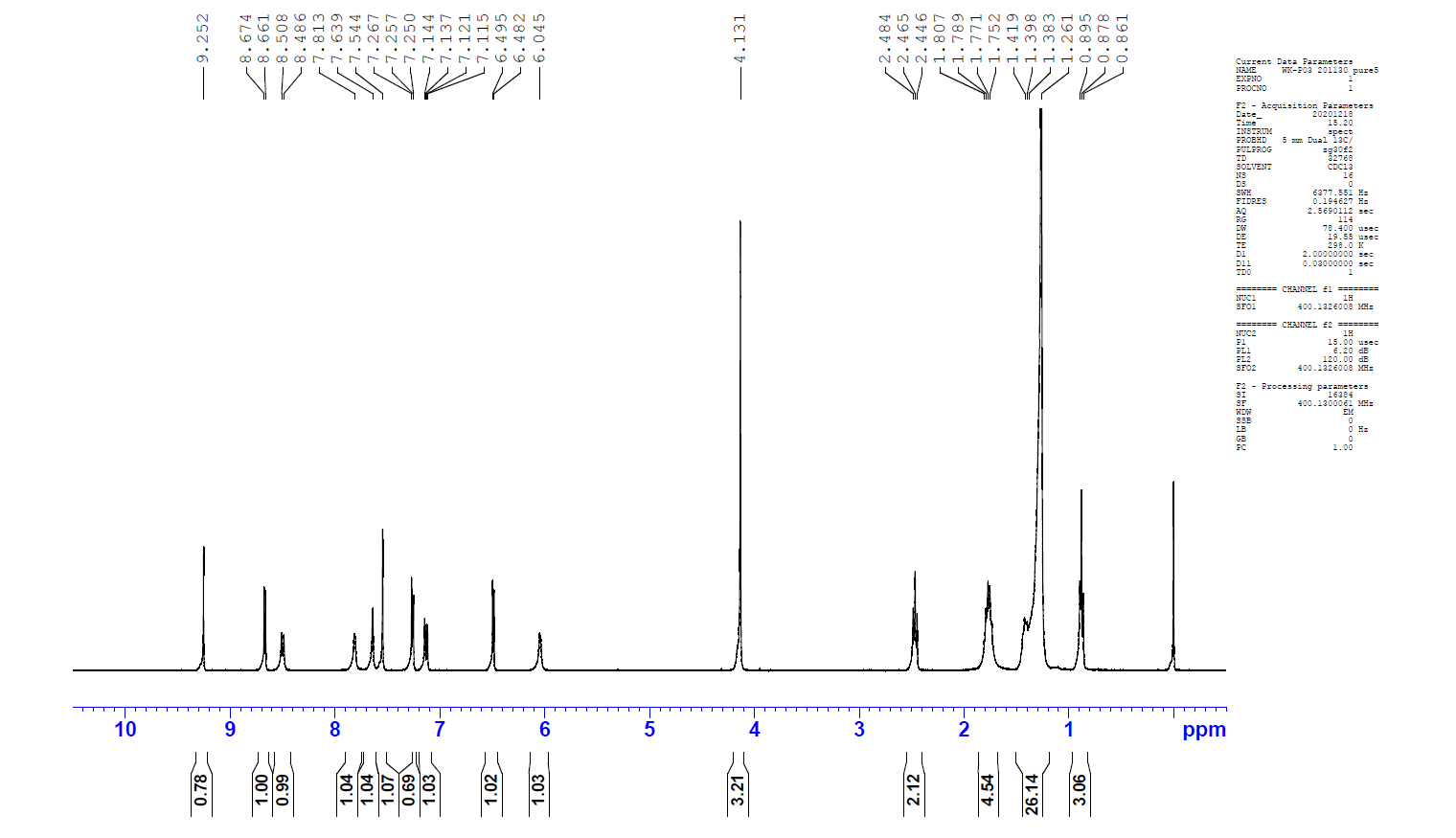

**Supplementary Figure 1.** The ^1^H-NMR spectrum of side product 1 from lenvatinib reaction.

**Supplementary Figure 2.** The mass spectrometry of side product 1 from lenvatinib reaction (positive and negative charge).


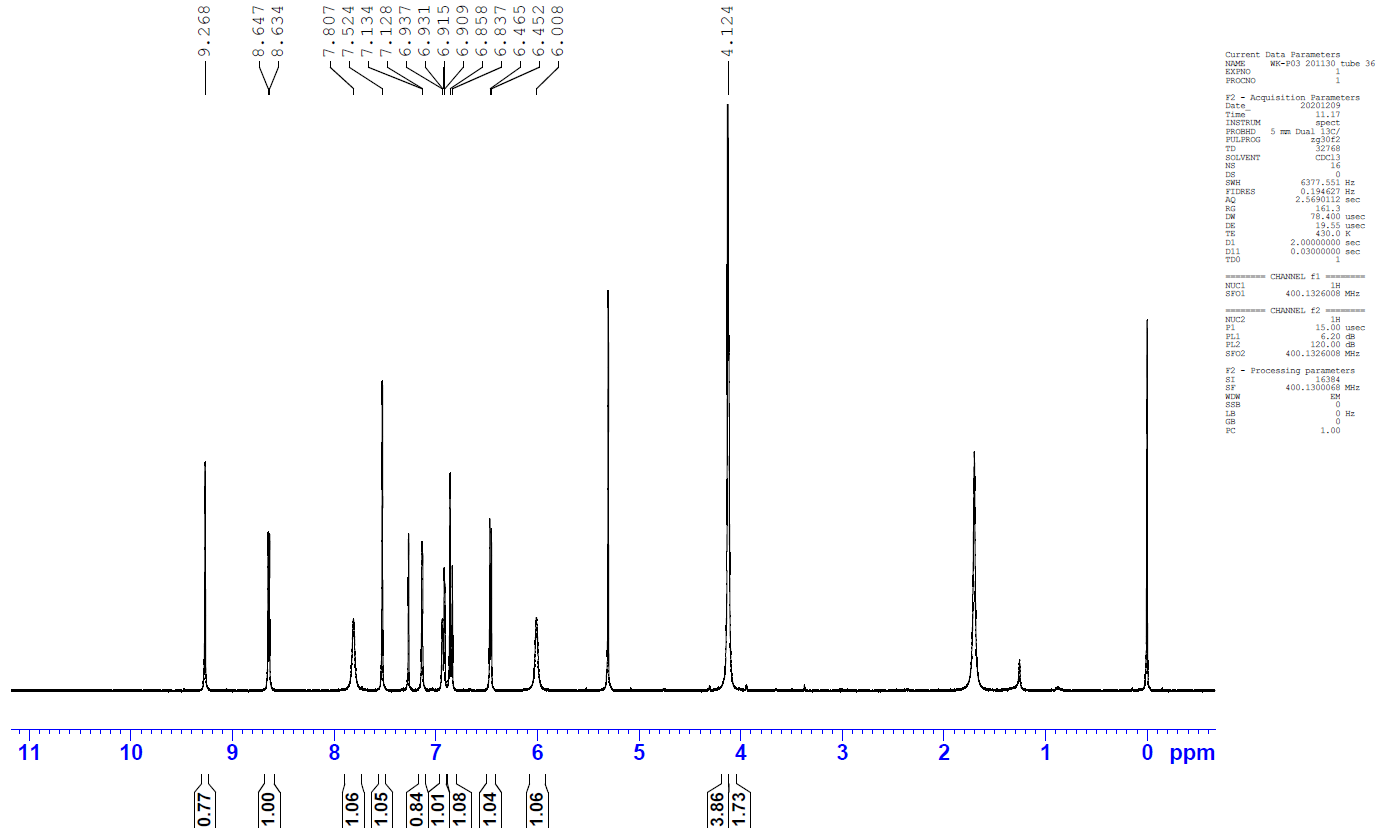

**Supplementary Figure 3.** The ^1^H-NMR spectrum of side product 2 from lenvatinib reaction.

**Supplementary Figure 4.** The mass spectrometry of side product 2 from lenvatinib reaction (positive and negative charge).


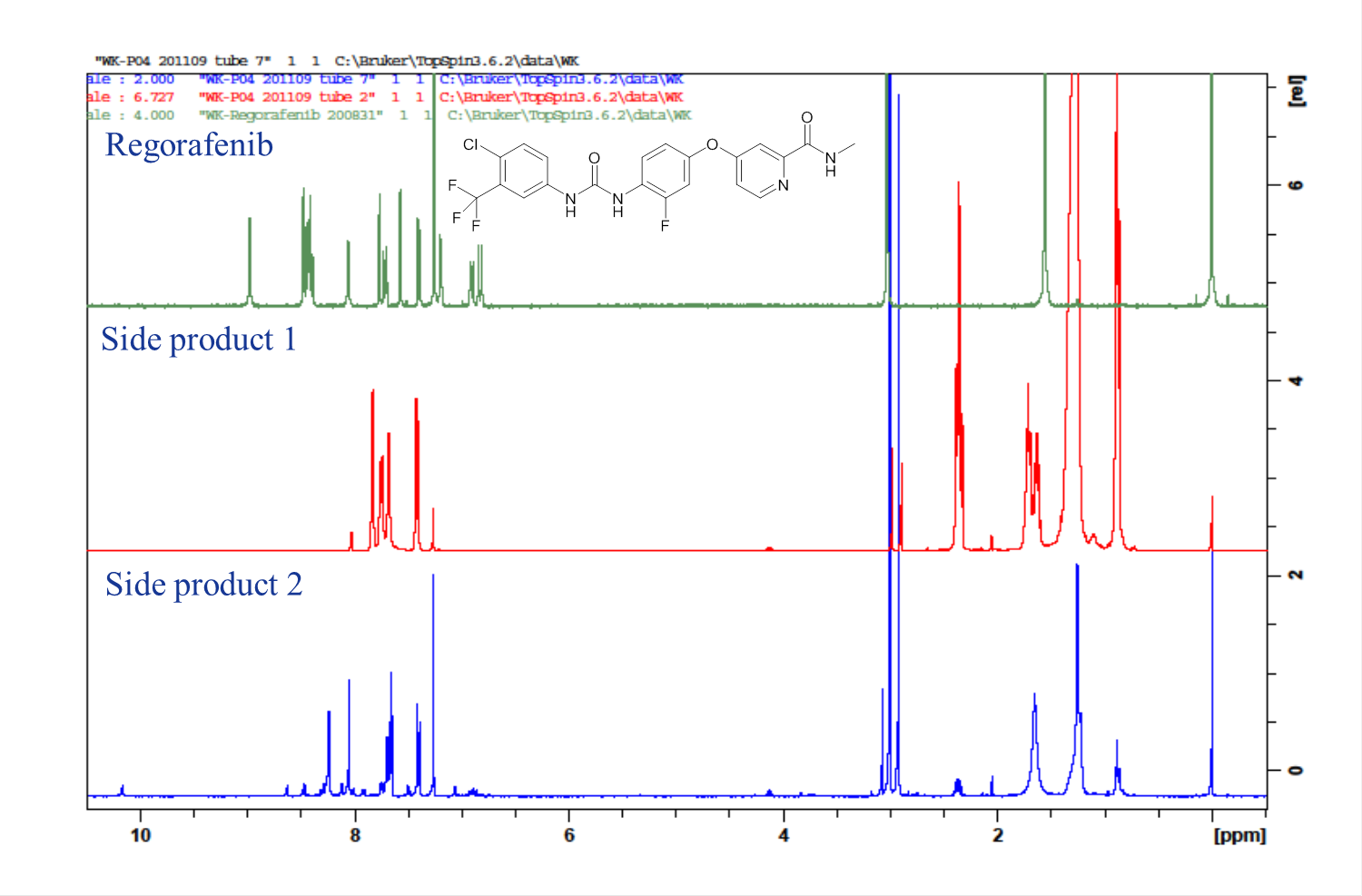


**Supplementary Figure 5.** The compared ^1^H-NMR spectrums in regorafenib reaction, two side products were found as fragmented structures.


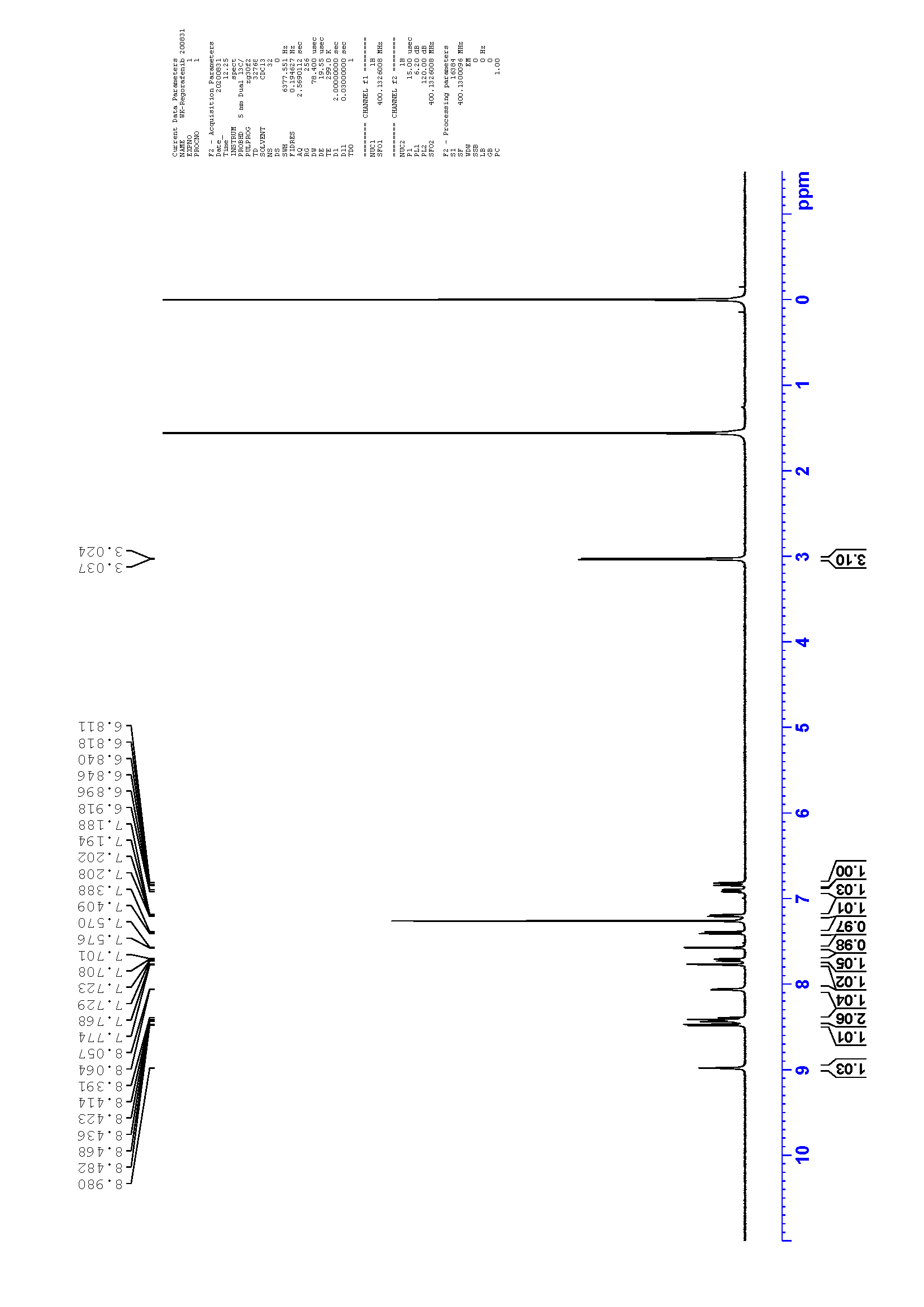


**Supplementary Figure 6.** The ^1^H-NMR spectrum of regorafenib.


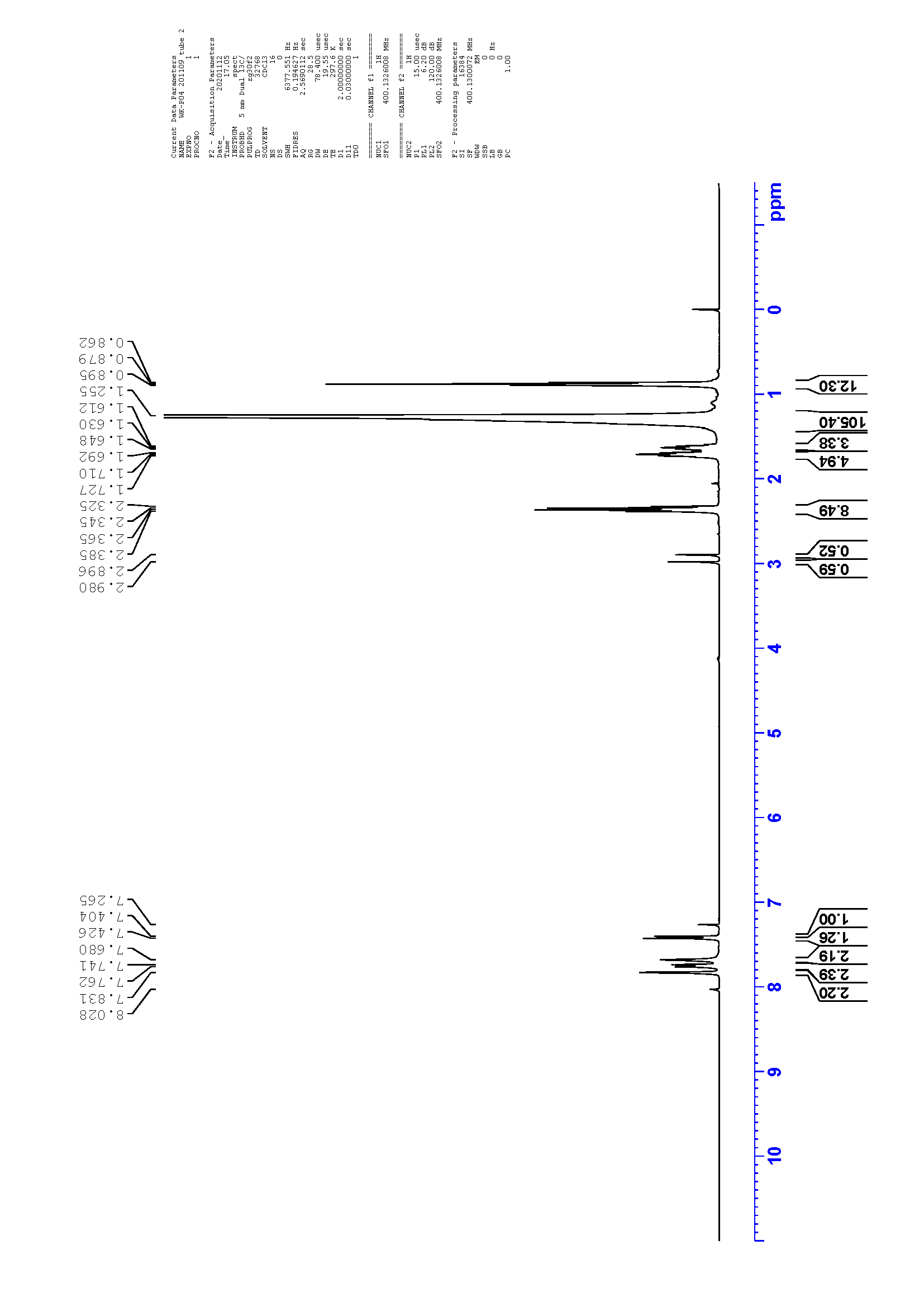


**Supplementary Figure 7.** The ^1^H-NMR spectrum of side product 1.


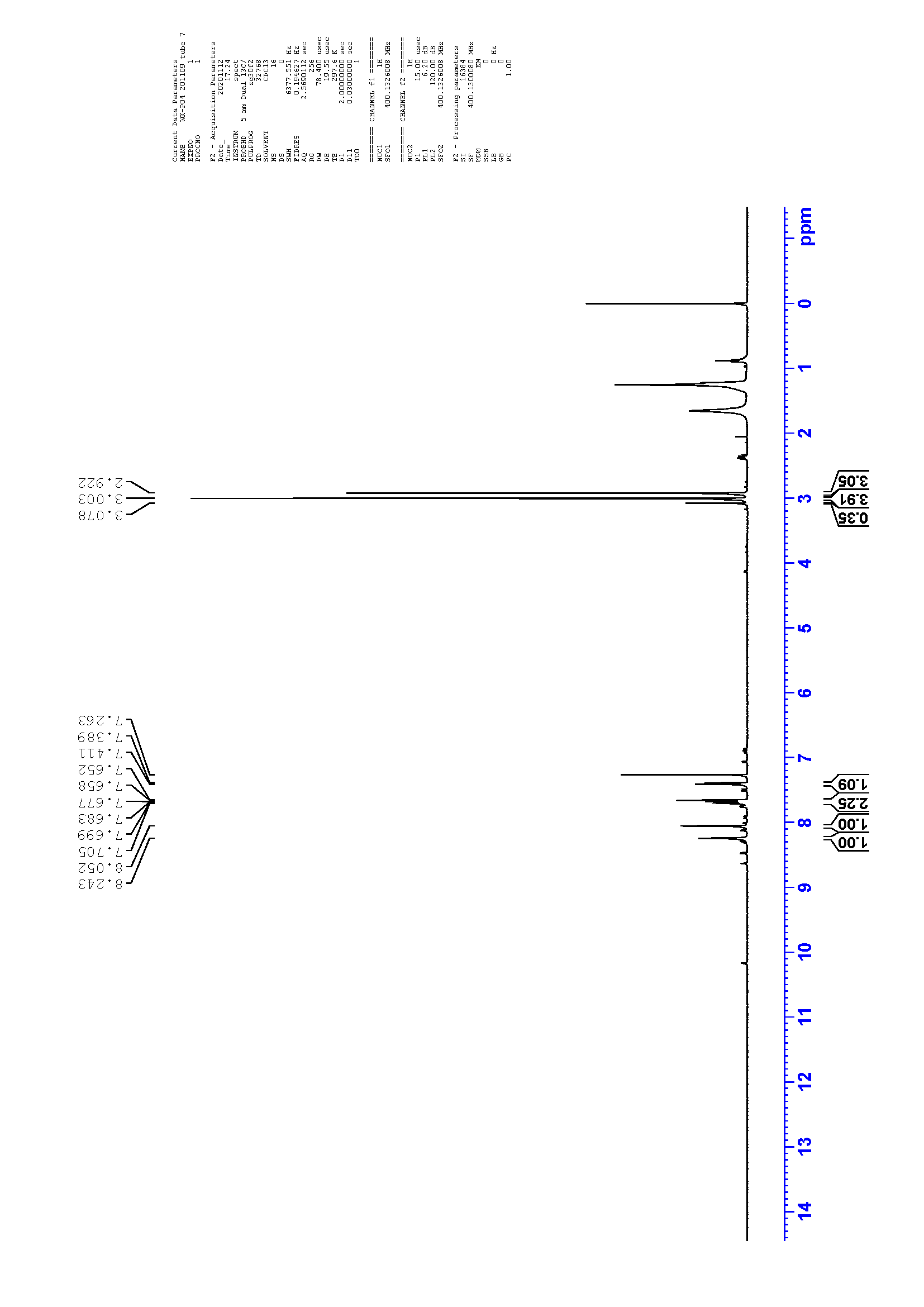


**Supplementary Figure 8.** The ^1^H-NMR spectrum of side product 2.


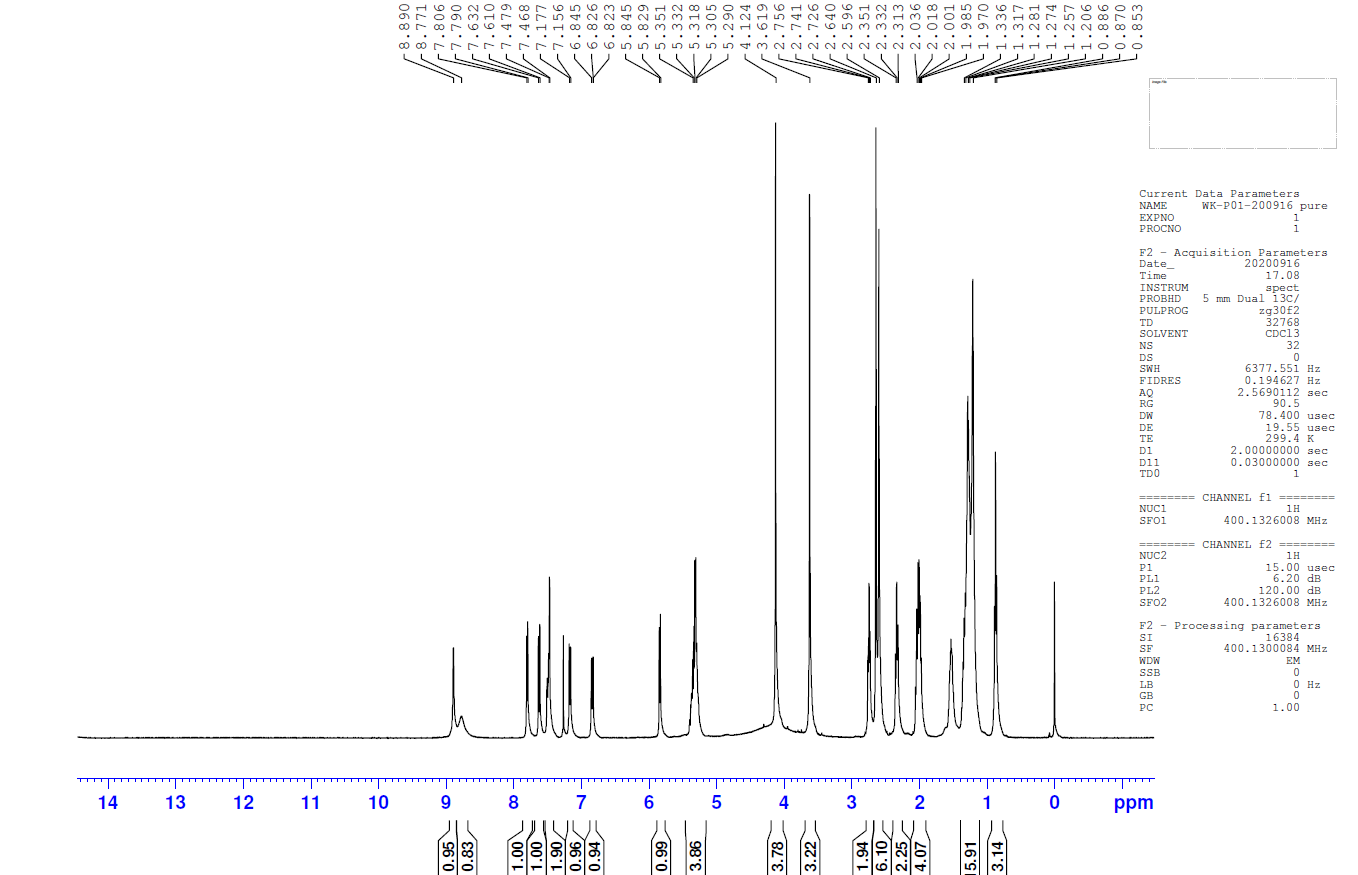

**Supplementary Figure 9.** The ^1^H-NMR spectrum of LAPC.


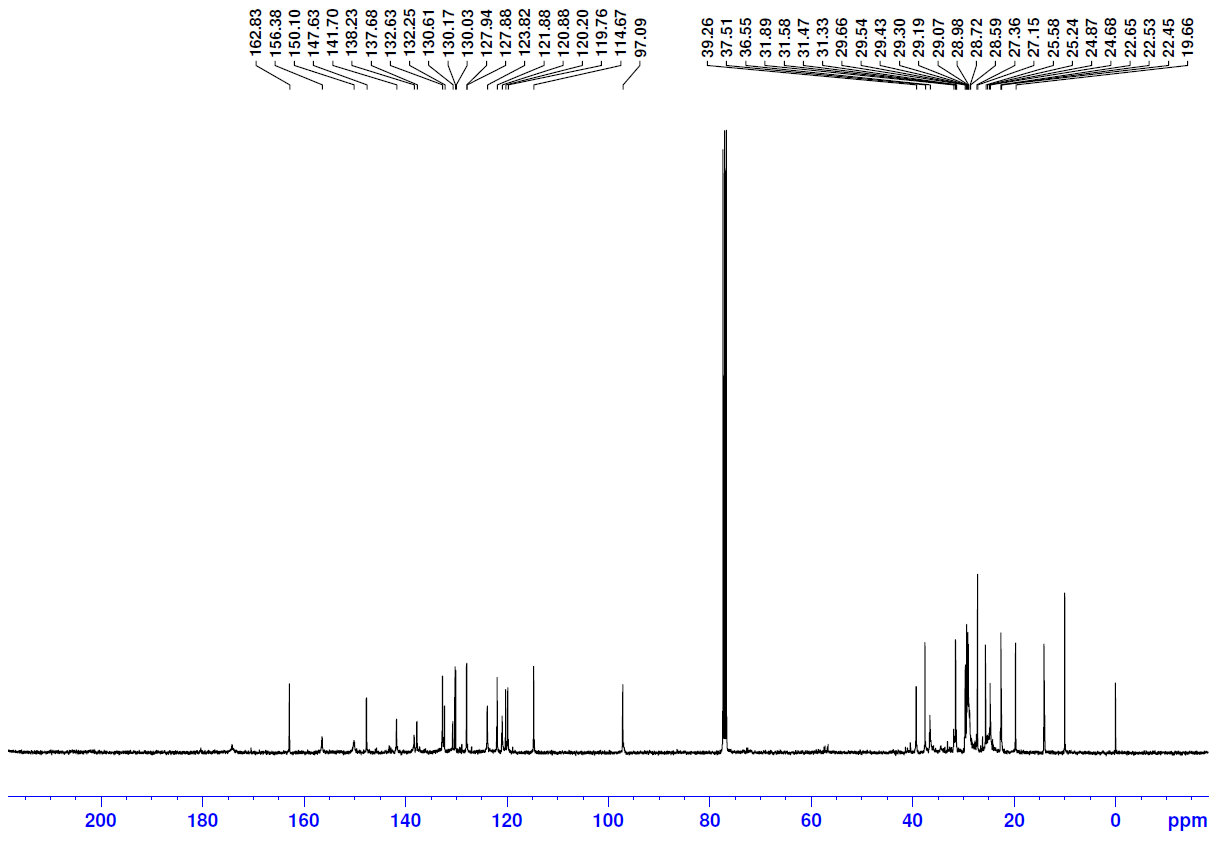

**Supplementary Figure 10.** The ^13^C-NMR spectrum of LAPC.

**Supplementary Figure 11.** The high resolution mass spectrometry of LAPC, [M+1]^+^.

**Supplementary Figure 12.** The high resolution mass spectrometry of LAPC, [M-1]^-^.
